# Social and ethical implications of AI-based facial technologies in medicine and healthcare: a scoping review

**DOI:** 10.1186/s12910-026-01564-7

**Published:** 2026-07-28

**Authors:** Helena Machado, Susana Silva, João Santos, Niklas Juth

**Affiliations:** 1 CIES-Iscte, University Institute of Lisbon, Lisbon, Portugal; 2 Institute of Social Sciences, Centre for Research in Anthropology (CRIA-UMinho/IN2PAST), University of Minho, Braga, Portugal; 3 CIES-Iscte, University Institute of Lisbon, Lisbon, Portugal; 4 Department of Public Health and Caring Sciences, Centre for Research Ethics & Bioethics (CRB), Uppsala University, Uppsala, Sweden

**Keywords:** Facial Recognition, Automate Facial Recognition, Bioethical Issues, Social Justice, Artificial Intelligence, Delivery of Health Care, Interdisciplinary Communication

## Abstract

**Background:**

AI-based facial technologies are increasingly explored in medicine and healthcare for purposes ranging from diagnosis and monitoring to identification. While ethical debates on AI-in-health are well developed, the specific social and ethical implications of facial AI in medical and healthcare contexts remain fragmented.

**Methods:**

We conducted a scoping review following PRISMA-ScR guidelines to map the social and ethical implications of AI-based facial technologies reported in the literature and to examine how these implications are framed across disciplinary domains. Searches of PubMed and Web of Science up to 9 October 2025 identified 43 peer-reviewed articles that met the inclusion criteria. Data were extracted using a standardised template and synthesised through content analysis.

**Results:**

Most articles were published after 2020 and were empirical in nature. The most frequently discussed issues concerned safety, clinical risk, privacy, surveillance, data governance, autonomy, and consent. These concerns largely align with established AI-in-health ethics and mainstream bioethical frameworks. Substantive engagements with social justice, stigma, appearance norms, and patient and public perspectives were less prominent. Ethical framings varied across disciplinary domains, with technical and clinical fields tending to adopt instrumental approaches, while bioethics, legal studies, and social sciences more often addressed broader sociopolitical implications.

**Conclusion:**

The literature on AI-based facial technologies in medicine and healthcare largely reproduces generic AI-in-health ethical concerns, while under-analysing face-specific moral stakes linked to identity, visibility, stigma, and vulnerability. Addressing these gaps requires more context-sensitive interdisciplinary ethical approaches that integrate justice broadly conceived and incorporate patients and public perspectives into ethical governance.

**Supplementary Information:**

The online version contains supplementary material available at https://doi.org/10.1186/s12910-026-01564-7.

## Background

Current applications of AI-based facial technologies in medicine and healthcare remain largely confined to experimental studies and pilot deployments, with systematic reviews and clinical trials highlighting technical, ethical and regulatory barriers to broader implementation [1–4]. At the same time, technical, clinical and ethics literatures repeatedly describe these technologies as prospective tools for routine screening, diagnosis, monitoring and patient identification in everyday care [2, 5–8].

In this article, we use the term AI-based facial technologies to refer to systems that either recognise individuals by matching their faces to stored templates (facial recognition) or analyse facial features, expressions or movements to infer information such as disease risk, emotional state or treatment adherence (facial analysis) [2, 9]. These systems are currently explored or deployed for a wide range of purposes, including rare-disease diagnosis through automated facial phenotyping, psychiatric and neurological assessment, aesthetic and reconstructive surgery planning, monitoring of medication adherence, and health-related smart-city surveillance [2, 5, 8, 10–12].

Faces are not “just another” biometric. They are central to personal identity and social recognition, and closely tied to appearance, stigma and social sorting [13, 14]. In medicine and healthcare, AI-based facial technologies operate precisely at this intersection. They promise earlier and more “objective” diagnosis of genetic or neurological conditions, novel tools for mental-health assessment and behavioural monitoring, optimisation of facial aesthetics and reconstructive outcomes, and more efficient identification or triage of patients in clinical and public-health settings [2–8, 18–21]. At the same time, they raise concerns about bias and discrimination, privacy and surveillance, consent and data governance, commercial exploitation of facial data [5, 9–11, 13–16, 18], and the potential dehumanisation of care [22].

Social and ethical debates on AI in medicine and healthcare and on facial recognition more broadly are already extensive. Work in AI ethics has examined issues such as fairness, bias and representativeness of training data; privacy and data protection; transparency and explainability; accountability and liability; and the impact of automation on professional roles and patient–clinician relationships [5, 10–12, 15, 22]. At the same time, there are common grounds to healthcare ethics discussions regarding clinical risk-benefit balance related to false positives and negatives, and under- and overtreatment [23], as well as the pathologisation or medicalisation of phenotypic facial traits [24]. In parallel, legal and policy literatures have analysed facial recognition as a particularly controversial technology, with a focus on surveillance, policing, human rights and democratic oversight [9, 13, 16–18]. Together, these strands position AI-based facial technologies as ethically distinctive within the wider landscape of data processing [5, 9, 11, 13–15, 18, 22]. However, the specific ethical implications of facial AI in medicine and healthcare have not been examined systematically, particularly in contexts where these systems are embedded in clinical decision-making, patient care and public-health infrastructures.

Existing reviews tend to follow two main paths. Some are primarily technical, concentrating on performance metrics such as accuracy, robustness and generalisability of algorithms [1–3, 19–21]. Others address the ethics of AI in medicine and healthcare in general, where facial technologies appear as one example among many, and rarely examined in depth as a face-based modality [10, 12, 15, 18, 22]. As a result, most ethical analyses apply broad AI-ethics checklists [15, 25] while paying limited attention to the specific moral stakes of facial AI. To address this gap, we conduct a scoping review that maps which social and ethical implications of AI-based facial technologies in medicine and healthcare are reported in the literature, and how these implications are framed across different disciplinary domains.

## Methods

This scoping review followed the methodological framework proposed by Peters et al. [26] and the PRISMA Extension for Scoping Reviews (PRISMA-ScR) checklist and explanation [27]. Our research question was: *What social and ethical implications of AI-based facial technologies are reported in the literature on medicine and healthcare*,* and how are these implications framed across disciplinary domains?*

**Fig. 1:**
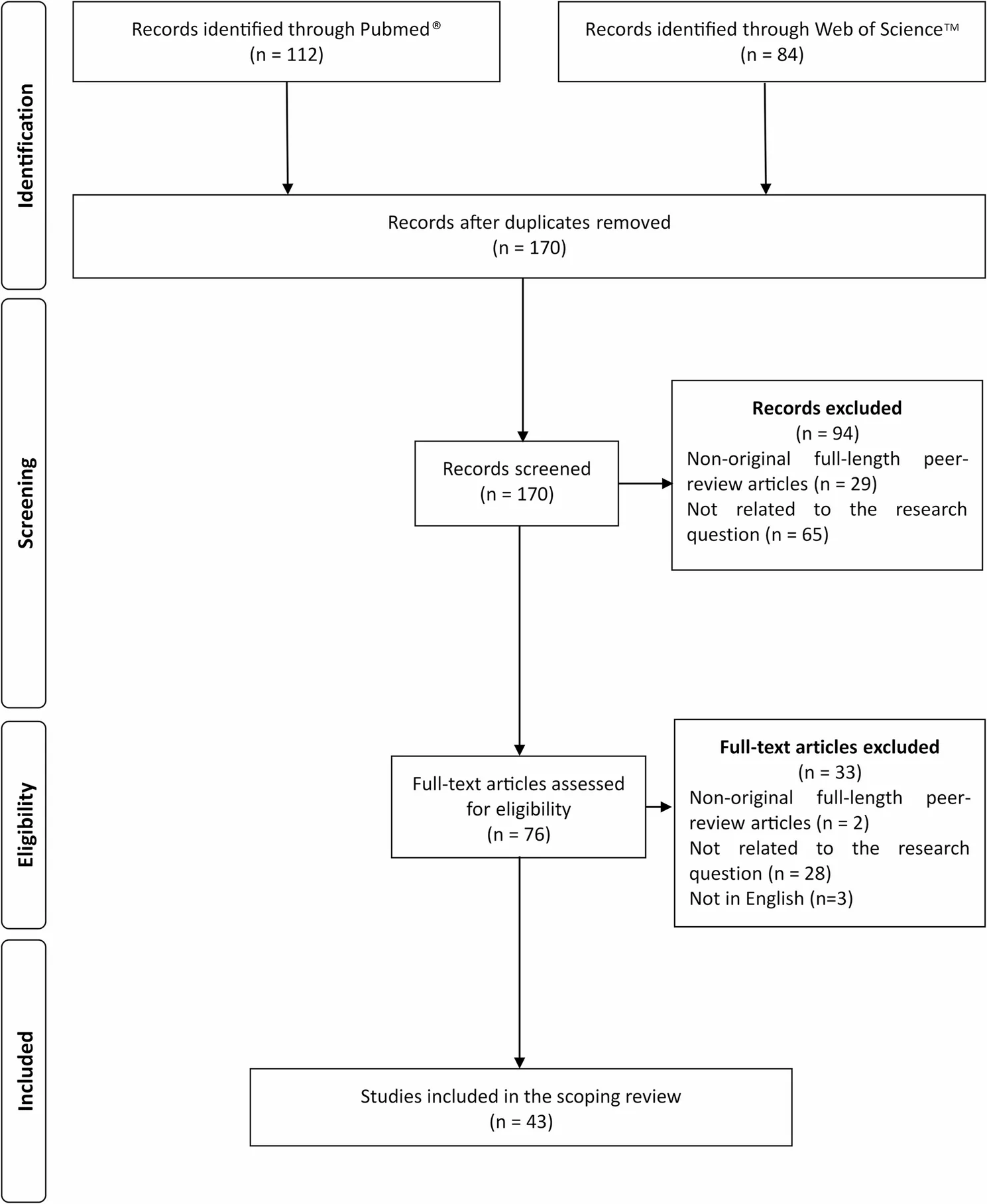
Flowchart showing the search results and screening process

Literature searches up to 9 October 2025 were conducted in PubMed^®^ and Web of Science™, using the following search expression: (“facial recognition” OR “face recognition” OR “facial expression recognition” OR “facial analysis”) AND (ethic* OR bioethic*) AND (medicine OR health*). No language or timeframe filters were applied at the search stage. The subsequent study selection process is summarised in Fig. 1.

Eligible publications were peer-reviewed, full-length journal articles in English that contained substantive discussion of social and/or ethical implications of AI-based facial technologies within medicine or healthcare. We excluded systematic reviews and meta-analyses, purely technical or engineering-focused articles, studies involving non-humans (animals or robots) or focusing on forensic or criminal justice applications without a clear health dimension. A total of 43 articles met the inclusion criteria.

Two authors (SS and HM) independently screened all the articles retrieved initially, based on the title and abstract, and afterward, based on the full text. At each stage, discrepancies were discussed and resolved by consensus.

The variables extracted, according to a standardised form, are presented in Additional file 1. The data-extraction template was developed by two authors (SS and HM) and then applied to included articles in two distinct stages.

In the first stage, data were extracted into all pre-specified categories defined by the authors, using a Python script and a large language model (LLM) developed by JS (GPT-5 mini via the OpenAI API; full prompt available in additional file 2). The model was designed to be fully aligned with emerging professional and editorial standards for AI use in scholarly research and publishing, as reflected in recent guidance from publishers and organisations such as the International Committee of Medical Journal Editors, by ensuring transparency, appropriate disclosure of AI use, and author responsibility. Thus, in the second stage, independent manual full-text review, coding, validation and correction by the first two authors before being charted in MS Excel (see Additional file 3). In cases where the model had omitted or mischaracterized data, these were identified, rectified or expanded during the second stage through full-text manual analysis. As the initial data charting was performed with the assistance of a LLM, whose outputs can vary between runs, this step is not fully reproductible.

Descriptive data for the characterization of articles included information about the authors and year of publication, the type of study (philosophical, empirical, other), the AI-based facial technology under analysis and its main functions in the healthcare context. Data on whether the ethical and social issues were identified in general terms, mentioned with some reflection or analysed in-depth were retrieved.

For each article, we identified the one or two disciplinary domains that most strongly frame and support its argument. Domains were selected from: clinical medicine/surgical specialties; psychiatry/psychology/neuroscience; public health/nursing/population health; computer science/engineering/AI; law/regulation/data protection; bioethics/philosophy/ethics; and social sciences/STS/humanities.

Data on the key ethical documents cited in each paper were also gathered and then grouped into five categories: data protection and AI governance frameworks; technical or clinical norms/guidelines; theoretical or philosophical approaches; reports, campaigns, media, documents from NGOs; and institutional procedures and approvals. Authors’ accounts of the social and ethical implications of AI-based facial technologies on medicine and healthcare were identified based on the content analysis technique, and synthesized into the following categories: bias/discrimination/inequity; privacy/confidentiality/surveillance; consent/autonomy; accountability/liability; stigma/labelling/stereotyping; dehumanisation/relational aspects of care; data governance and secondary use; safety/technical robustness/clinical risk/harm; trust/acceptability; justice; human agency and oversight; transparency; other. Categories were initially derived from existing AI-in-health ethics literature and then iteratively refined during coding. The incorporation of an open-ended category and a comprehensive manual review served as mitigation strategies to avert the under-identification of novel emergent social and ethical implications of AI-based facial technologies in medicine and healthcare.

**Fig. 2:**
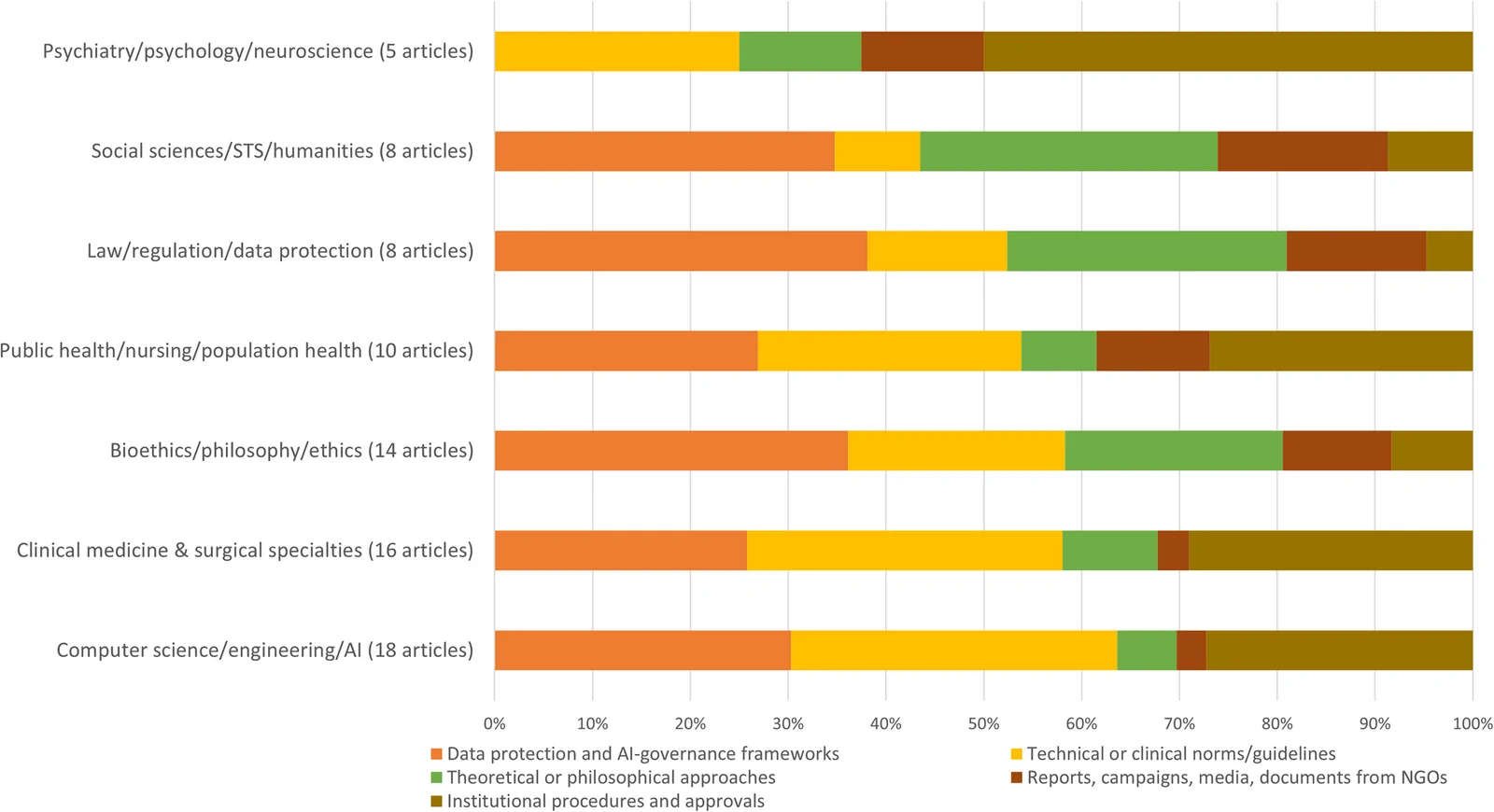
Ethical frameworks, according to the main disciplinary domains of each article

**Fig. 3:**
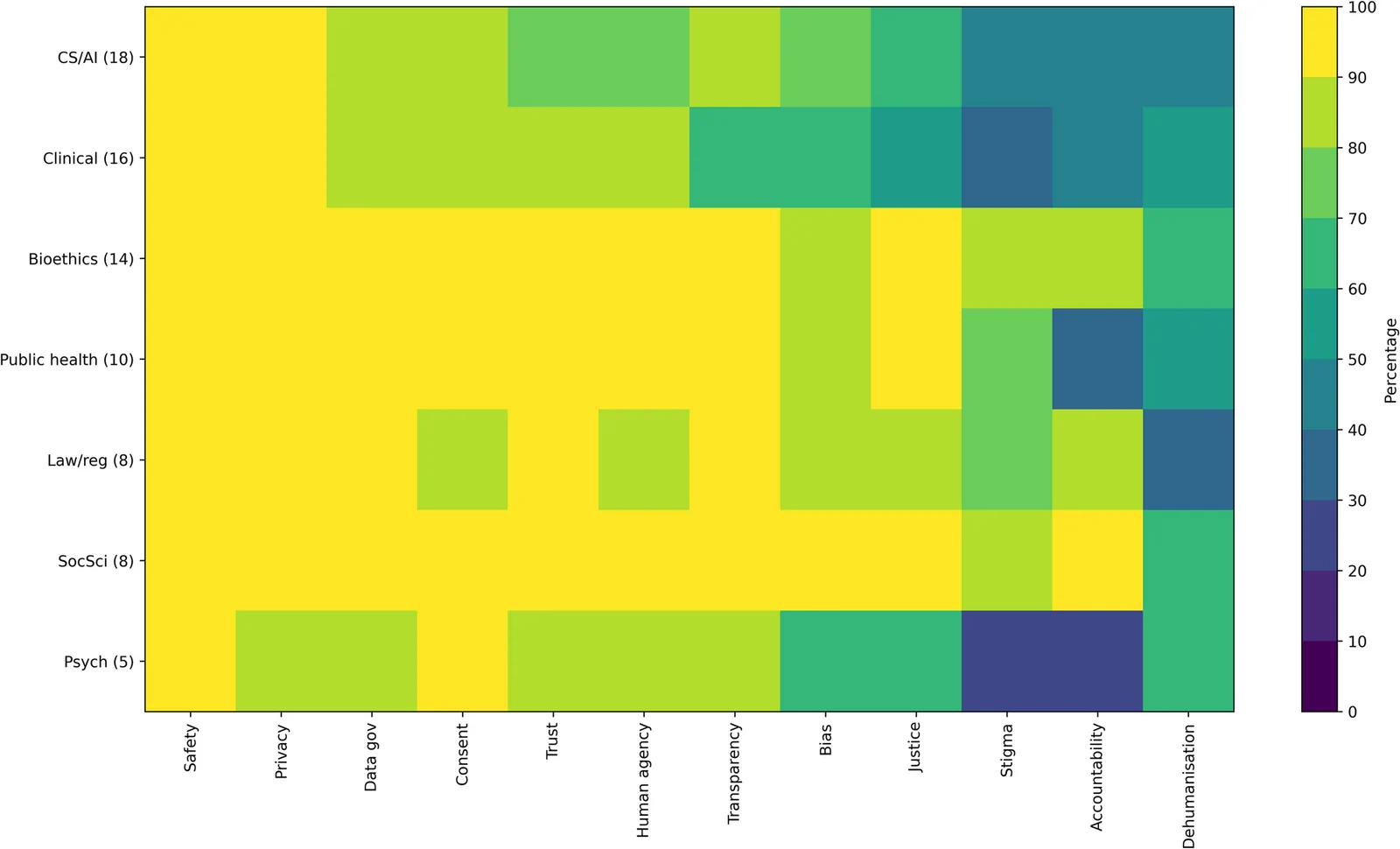
Social and ethical implications of AI-based facial technologies in medicine/healthcare, according to disciplinary domains

The main characteristics of the 43 studies included can be found in Table 1. Articles were grouped by type of study and ordered by the year of publication. The main disciplinary domains and the ethical frameworks of each article are presented in Fig. 2. The social and ethical implications of AI-based facial technologies in medicine and healthcare are summarised in Table 2 with illustrative quotes selected by the authors during the manual review phase, and cross-referenced with the relevant disciplinary fields in Fig. 3. The results are presented below in a narrative format, with complementary tables and figures to provide a visual representation of key findings.

**Table 1:** Main characteristics of the studies included in the scoping review (*n* = 43)

	AI-based technology	Main functions in healthcare	Mode of presenting social and ethical issues
Empirical articles (*n* = 25)			
Roberts, Troiano, Spiegel, 2016 [34]	Facial recognition	Identify individuals; Other (medical publication/education)	Analyzed in-depth
Ip, Barnard, Marshall, et al., 2017 [28]	Facial recognition	Diagnosis/screening; Research-only tool (dataset/method development)	Analyzed in-depth
Sero, Zaidi, Li, et al., 2019 [40]	Facial recognition	Identify individuals; Research-only tool (dataset/method development)	Some refletion
Costa, Pires, Sousa, et al., 2021 [31]	Facial analysis	Risk prediction/prognosis; Monitoring/telemedicine; Research-only tool (dataset/method development)	Some refletion
Franco, Oneto, Navarin, et al., 2021 [41]	Facial recognition	Identify individuals; Research-only tool (dataset/method development)	Analyzed in-depth
Hayashi, Kamezaki, Sugano, 2021 [42]	Facial analysis	Risk prediction/prognosis; Diagnosis/screening; Monitoring/telemedicine; Research-only tool (dataset/method development)	Some refletion
Marwaha, Chitayat, Meyn, et al., 2021 [35]	Facial analysis	Diagnosis/screening; Triage/workflow/administration; Other (medical education)	Some refletion
Seto, Hosaka, Ishida, et al., 2021 [43]	Facial analysis	Monitoring/telemedicine; Triage/workflow/administration	Some refletion
Brooks, Nguyen, Bhatti, et al., 2022 [36]	Both	Monitoring/telemedicine; Other (marketing and consumer behaviour influence)	Some refletion
Katsanis, Claes, Doerr, et al., 2022 [37]	Both	Research-only tool (dataset/method development); Other (assessment of public views)	Some refletion
Morita, Shirai, Shinkoda, et al., 2022 [30]	Facial analysis	Monitoring/telemedicine; Research-only tool (dataset/method development)	Some refletion
Rendell, Schmidt, Neergaard, et al., 2022 [44]	Facial analysis	Monitoring/telemedicine; Triage/workflow/administration	Analyzed in-depth
Boutin, Lévêque, Desmoulin-Canselier, 2023 [45]	Facial analysis	Diagnosis/screening; Monitoring/telemedicine; Triage/workflow/administration	Analyzed in-depth
Hallowell, Badger, McKay, et al., 2023 [46]	Facial analysis	Diagnosis/screening; Triage/workflow/administration	Analyzed in-depth
Lin, Zhou, Chen, et al., 2024 [38]	Facial analysis	Diagnosis/screening; Monitoring/telemedicine; Triage/workflow/administration	Some refletion
Tutuianu, Liu, Alamaki, et al., 2024 [47]	Facial analysis	Diagnosis/screening; Monitoring/telemedicine; Research-only tool (dataset/method development)	Analyzed in-depth
Wang, Zhang, Kang, et al., 2024 [33]	Facial analysis	Monitoring/telemedicine; Other (emotional support)	Some refletion
Al Menabbawy, Ruhser, El Refaee, et al., 2025 [48]	Facial analysis	Risk prediction/prognosis; Diagnosis/screening; Monitoring/telemedicine	General terms, without detailed analysis
Ali, Cui, 2025 [49]	Facial analysis	Research-only tool (dataset/method development)	Some refletion
Ali, Cui, 2025 [50]	Facial analysis	Diagnosis/screening; Triage/workflow/administration	Some refletion
Amado, Karataş, Yüvrük, et al., 2025 [29]	Facial recognition	Identify individuals; Research-only tool (dataset/method development)	Some refletion
Ferreira, Marinheiro, Mateus, et al., 2025 [51]	Both	Risk prediction/prognosis; Monitoring/telemedicine; Triage/workflow/administration	Analyzed in-depth
Tran, Dang, Nguyen, et al., 2025 [52]	Facial recognition	Identify individuals; Monitoring/telemedicine	Analyzed in-depth
Wagner, Mancuso, Stoner, et al., 2025 [53]	Facial recognition	Monitoring/telemedicine; Triage/workflow/administration	Analyzed in-depth
Wang, Hu, Xu, et al., 2025 [54]	Facial analysis	Diagnosis/screening	Some refletion
Philosophical articles (*n* = 8)			
Van Biesen, Decruyenaere, Sideri, et al., 2019 [39]	Facial recognition	Risk prediction/prognosis; Monitoring/telemedicine	Analyzed in-depth
Toribio-Roura, 2020 [55]	Facial recognition	Identify individuals; Monitoring/telemedicine; Triage/workflow/administration	Analyzed in-depth
Moraes, Almeida, Pereira, 2021 [56]	Facial recognition	Identify individuals; Monitoring/telemedicine	Analyzed in-depth
Fontes, Hohma, Corrigan, et al., 2022 [57]	Facial recognition	Risk prediction/prognosis; Identify individuals; Monitoring/telemedicine; Triage/workflow/administration	Analyzed in-depth
Li, Hua, Sun, et al., 2022 [58]	Facial recognition	Identify individuals; Monitoring/telemedicine	Analyzed in-depth
Raposo, Du, 2023 [59]	Facial recognition	Identify individuals; Monitoring/telemedicine	Analyzed in-depth
Raposo, 2024 [18]	Both	Risk prediction/prognosis; Diagnosis/screening; Identify individuals; Monitoring/telemedicine; Triage/workflow/administration; Research-only tool (dataset/method development)	Analyzed in-depth
Rajendra, Thuraisingam, 2025 [60]	Both	Risk prediction/prognosis; Diagnosis/screening	Analyzed in-depth
Other type of articles (*n* = 10)			
Martinez-Martin, 2019 [5]	Both	Risk prediction/prognosis; Diagnosis/screening; Identify individuals; Monitoring/telemedicine; Research-only tool (dataset/method development)	Analyzed in-depth
Vidanaarachchi, Seneviratne, Kasthuriarachchi, et al., 2021 [61]	Facial recognition	Identify individuals; Monitoring/telemedicine; Triage/workflow/administration	Some refletion
Su, Cheshmehzangi,‘McDonnell, et al., 2022 [62]	Facial recognition	Risk prediction/prognosis; Identify individuals; Monitoring/telemedicine; Triage/workflow/administration	Some refletion
Mir, Maurya, 2023 [63]	Both	Risk prediction/prognosis; Triage/workflow/administration	Some refletion
Hartmann, Rubeis, Primc, 2024 [32]	Facial analysis	Risk prediction/prognosis; Monitoring/telemedicine	Analyzed in-depth
Khan, Topham, Khayam, et al., 2024 [64]	Both	Risk prediction/prognosis; Identify individuals; Monitoring/telemedicine	Analyzed in-depth
Al-Dhubaibi, Mohammed, Atef, et al., 2025 [65]	Facial analysis	Risk prediction/prognosis; Diagnosis/screening; Monitoring/telemedicine; Triage/workflow/administration	Analyzed in-depth
Barone, De Bernardis, Persichetti, 2025 [66]	Facial analysis	Risk prediction/prognosis; Monitoring/telemedicine; Triage/workflow/administration	Some refletion
Lei, Dang, Song, et al., 2025 [2]	Facial analysis	Risk prediction/prognosis; Diagnosis/screening; Monitoring/telemedicine	Analyzed in-depth
Makhseed, Arian, Shuaib, 2025 [67]	Facial analysis	Risk prediction/prognosis; Triage/workflow/administration	Analyzed in-depth

**Table 2:** Social and ethical implications of AI-based facial technologies discussed in literature on medicine and healthcare

Safety/technical robustness/clinical risk/harm (42 articles) [2, 5, 18, 28–47, 49–67]: illustrative quotes
• There is thus a need for a strict and close regulation and/or oversight by an independent body, in order to protect patient and public safety and best interests [39]. • Despite the numerous benefits offered by AI in plastic surgery, several challenges remain including the need for robust data sets, algorithm validation, and integration into existing clinical workflows [66]. • Countering the promotion of products harmful to health will require monitoring and scrutiny of the actions of not only transnational food and beverage companies but also technology companies [36].
Privacy/confidentiality/surveillance (41 articles) [2, 5, 18, 28, 30–62, 64–67]: illustrative quotes
• Many of the respondents were positive about the use of cameras, despite concerns about the impact on privacy [43]. • This has raised concern about the right to privacy of individuals being identified: who is surveilling? In what context? For which purposes? [56] • Privacy concerns are critical challenges when deploying AI-assisted facial analysis for disease detection. (…) The collection, storage, and analysis of facial data raises significant concerns about the confidentiality and security of individuals’ personal information [2].
Data governance and secondary use (40 articles) [2, 5, 18, 28, 31–53, 55–67]: illustrative quotes
• Although marketed as ‘innovative’, the introduction of such systems raises questions as to the limitation of patient autonomy, secondary uses of patient data, impact on the physician’s liability, and artificial inflation of drug prices [39]. • Although the prevalence of facial recognition-based COVID-19 surveillance tools and techniques, China does not have a facial recognition law to protect its residents’ facial data [62]. • FRT presents some ethical and legal challenges, such as (…) the risk of using this information for other purposes besides public health [59].
Consent/autonomy (39 articles) [2, 5, 18, 28–29, 31–42, 44–60, 62, 64–67]: illustrative quotes
• Patients must be made aware of how their facial data will be used, stored, and analyzed, and must provide explicit consent before any imaging or data collection takes place [50]. • This poses two types of challenges: risks at an individual’s level, which relate to personal privacy, and societal risks, involving the possibility of tracking each individual and making inferences about any person or group, having in profiling an emergent threat to individual autonomy and democratic values [57].
Trust/acceptability (38 articles) [2, 5, 18, 28, 31–35, 37–47, 49–62, 64–67]: illustrative quotes
• Ensuring that these images are securely stored and that data privacy is protected is essential for maintaining patient trust [50]. • The trust is established by looking, or basically believing or getting proof that the best technology is being used, but more important than that, that the data that’s being used to train the system is validated [46]. • Making face recognition algorithms more trustworthy (fair, private, and explainable) would greatly improve the public opinion of them and their general acceptance [41].
Human agency and oversight (37 articles) [2, 5, 18, 28, 31–42, 44–47, 49–53, 55–60, 62–67]: illustrative quotes
• Until AI’s achieve fairness, imaginative agency can be used to contest dubious algorithms such as those of face recognition systems [55]. • A worker-centric approach, grounded in data transparency, privacy protection, and ethical oversight, is essential to ensuring that video-based health monitoring remains a tool for supporting employees rather than evaluating them [51]. • There is thus a need for a strict and close regulation and/or oversight by an independent body, in order to protect patient and public safety and best interests [39].
Transparency (37 articles) [2, 5, 18, 29, 31–33, 35–47, 49–53, 55–65, 67]: illustrative quotes
• Fundamental rights (…) are daily undermined by the use of increasingly complex and less understandable yet more accurate learning algorithms [41]. • Transparency on the part of the developer of these issues has value in being able to confidently interpret results in clinical practice [35]. • Building patient trust in AI-supported aesthetic therapy relies on transparency about the contribution of algorithms to decision-making [65].
Bias/discrimination/inequity (33 articles) [2, 5, 18, 29, 31, 32, 35–41, 44–47, 49–52, 55–61, 63–67]: illustrative quotes
• To avoid potential algorithmic biases, one must properly represent all minority groups in the databases being queried [40]. • Commercial companies in the real world use facial recognition technology to develop products to meet people’s needs on the one hand and use facial recognition technology to obtain facial information of users or individuals for other purposes, such as better price discrimination [58]. • Increasing use of FRT is concerning due to its inaccuracies, which can exacerbate social inequalities, particularly in identifying communities of colour [64].
Justice (distributive/epistemic) (32 articles) [2, 5, 18, 29, 31, 32, 35–41, 44–47, 49–53, 55–60, 62, 64, 65, 67]: illustrative quotes
• Model unfairness in AI-assisted facial analysis for disease detection arises when AI models perform differently across demographic groups due to biases in their training datasets, a situation exacerbated by a lack of diversity in ethnicity, sex, and age [2]. • [The paper] identifies key challenges such as limited enforcement mechanisms, legal ambiguity, the trade-off between accuracy and interpretability, and cross-jurisdictional inconsistencies. The paper concludes with policy recommendations that include strengthening statutory obligations for explainability, establishing algorithmic audit frameworks, developing transparency registries, and promoting greater international coordination in AI governance [60]. • It is imperative that we seek a better understanding of individuals’ informational needs and facilitate opportunities for individuals to develop informed opinions on these issues before, in the absence of a collective will, policymakers—or, more likely, corporations—make normative decisions that will be difficult to change or reverse once established [37].
Stigma/labelling/stereotyping (25 articles) [2, 5, 18, 32, 36, 37, 39–41, 44–47, 51, 53, 55–61, 64, 65, 67]: illustrative quotes
• Machine learning techniques lead to a normalisation of the data: Larger deviations from the norm get discarded to enable algorithmic calculations, and the majority label continues to be used in further processing in the case of unclear data [32]. • Despite documented biases in the poor performance of FRT in identifying individuals who are not White men (…), racial and ethnic background had no significant effect on the perceived effectiveness of FRT in assessing race or ethnicity [37].
Accountability/liability (24 articles) [2, 5, 18, 33–35, 37, 39–41, 45–47, 52, 55–60, 62, 64, 65, 67]: illustrative quotes
• The inherent opacity of complex AI models, often described as the ‘black box problem,’ raises significant challenges in ensuring accountability, fairness, and due process when automated decisions impact individual rights and opportunities [60]. • If FRT diagnostic software develops to the point that it is used not just to augment but to replace a physician’s judgment, ethical and legal questions may arise regarding which entity appropriately has liability [5].
Dehumanisation/relational aspects of care (22 articles) [5, 30–33, 38, 39, 44–47, 49–51, 55, 56, 58, 60, 63–65, 67]: illustrative quotes
• The use of FRT in health care suggests the importance of (…) effective communication about incidental findings, and potential influence on patient-clinician relationships [5]. • Overreliance on AI may diminish the human-centric approach essential in aesthetic procedures, where patient expectations and subjective perceptions of beauty play a crucial role [65].

## Results

### Characteristics of the studies

The articles that met the inclusion criteria were published between 2016 and 2025. The publication activity is strongly skewed towards the last five years: only six articles were published up to 2020, whereas 37 (86%) appeared from 2021 onwards. A significant proportion of the articles were empirical studies (*n* = 25), while eight papers focused on philosophical or conceptual analyses and six synthesised evidence through narrative or comprehensive reviews. The majority of empirical studies (15/25) discussed facial analysis, whereas philosophical or conceptual articles are oriented toward facial recognition (6/8 articles). A thorough analysis of the ethical and social issues was conducted in all philosophical articles; however, this was only the case for two-fifths of the empirical studies (10/25). These publication characteristics suggest a recent and growing interest in AI-based facial technologies in medicine and healthcare, with a substantial proportion of work taking the form of empirical, data-driven studies.

### Functions of AI-based facial technologies in medicine and healthcare

Almost all studies covered overlapping functions. The majority of articles (*n* = 29) addressed subjects related to monitoring/telemedicine (e.g. public health surveillance, sleep-state and medication adherence monitoring), followed by triage/workflow/administration (*n* = 18; e.g. plastic surgery planning, support nursing observation), risk prediction/prognosis (*n* = 17; e.g. predict treatment outcomes in hemifacial spasm, in-vehicle safety intervention to prevent accidents), diagnosis/screening (*n* = 15; e.g. facial symmetry and proportions in aesthetic evaluations, rare diseases diagnosis, nutritional and functional assessment), and identification of individuals (*n* = 15; e.g. masked facial recognition, remote biometric identification). A total of twelve articles described research tools related to the following areas: the assessment of cognition in older adults [28]; face memory tests [29]; neonatal alertness state classification [30]; and assistance to women victims of violence [31], amongst others. A paucity of studies had addressed the social and ethical implications of AI-based facial technologies in behavioural intervention/therapy [32], emotional support [33], medical education/publication (e.g. guidelines for patient photograph deidentification) [34, 35], and marketing and consumer behaviour influence by leading global food and beverage companies [36]. One article explored U.S. adult perspectives on facial images [37]. This combination of clinical, administrative and security-related uses illustrates how facial AI in medicine and healthcare settings can be entangled with broader logics of surveillance, commercialisation and behavioural governance.

### Disciplinary domains and ethical frameworks

The articles encompassed a broad spectrum of disciplinary domains, albeit with an uneven distribution: computer science/engineering/AI (*n* = 18), clinical medicine and surgical specialties (*n* = 16) and bioethics/philosophy/ethics (*n* = 14) were predominant, followed by public health/nursing/population health (*n* = 10), law/regulation/data protection (*n* = 8) and social sciences/STS/humanities (*n* = 8). A smaller number of articles framed their analysis within psychiatry/psychology/neuroscience (*n* = 5). These results reveal a pattern that reflects the multidisciplinary nature of the debate surrounding the social and ethical implications of AI-based facial technologies in medicine and healthcare. However, the results also indicate that such discussion is often anchored in technical or clinical projects that subsequently involve ethical, legal or social analysis.

Figure 2 provides a visual representation of the distribution of ethical frameworks mobilised in the articles across the disciplinary domains. The findings reveal a heterogeneous distribution of ethical framings across disciplines. The fields of computer science/engineering/AI, clinical medicine/surgical specialties, and public health/nursing/population health are characterised primarily by technical or clinical norms/guidelines, illustrating the focus of these fields on metrics, standardisation and disciplinary governance. Data protection and AI governance frameworks, as well as institutional procedures and approvals were also present, but less prominent in computer science/engineering/AI, and clinical medicine/surgical specialties, reflecting the integration of regulatory and ethical issues into clinical and technical contexts. Data-protection, AI governance frameworks and theoretical or philosophical approaches were dominant in bioethics/philosophy/ethics, law/regulation and social sciences/STS/humanities. Furthermore, bioethics/philosophy/ethics is also oriented towards technical and clinical guidelines, reflecting an intersection between conceptual orientation and interest in regulatory and normative infrastructures. It is noteworthy that social sciences/STS/humanities displayed a broad spread across all ethical frameworks, including reports, campaigns, media and NGO documents, suggesting a plural epistemological landscape and engagement with civil-society and advocacy. Finally, the domain of psychiatry/psychology/neuroscience is anchored in institutional procedures and approvals. These patterns indicate disciplinary clustering in the ethical frameworks mobilised, with different fields drawing on partially distinct epistemic resources.

### Social and ethical issues

The social and ethical implications of AI-based facial technologies discussed in the literature on medicine and healthcare that met the inclusion criteria are depicted in Table 2. These implications were mapped by quantifying the number of studies that referred to each one. The most frequently cited issues in the literature were related to safety/technical robustness/clinical risk/harm (*n* = 42), privacy/confidentiality/surveillance (*n* = 41), data governance and secondary use (*n* = 40), consent/autonomy (*n* = 39), trust/acceptability (*n* = 38), human agency and oversight (*n* = 37), and transparency (*n* = 37). Other prevalent issues centre on bias/discrimination/inequity (*n* = 33) and distributive/epistemic justice (*n* = 32). The set of consistent core issues indicates a degree of consensus on fundamental ethical principles in medicine and healthcare. It also suggests that debates on facial AI largely mirror those in other contexts, where concerns about safety, privacy, governance, autonomy, and structural inequalities dominate. The reference to stigma, labelling and stereotyping is evident in 25 articles, including those addressing the potential of phenotype-based diagnostic tools and aesthetic assessment to reinforce narrow or racialised conceptions of normality and pathology, and their disproportionate reflection of Western beauty ideals. A total of 24 articles explore issues related to accountability/liability, including the development of guidelines for protecting patients in the context of facial photograph deidentification. In addition, the articles examine how contrasting regulatory approaches and political governance regimes deal with responsibility for errors in medicine and healthcare linked to facial AI. The dehumanisation and relational aspects of care are discussed in 22 articles, with a focus on the impacts on patient-provider relationships, the potential shift toward reductionist and mechanistic interpretations of emotions and behaviours, the erosion of healthcare providers’ skills, and the imaginative agency of ordinary citizens in the reconfiguration of the human dimension of healthcare. These concerns are predicated on the idea that the face is socially, culturally and emotionally loaded, thus giving rise to unique social and ethical implications that extend beyond those of other AI technologies. Issues such as industrial partnerships [35], commercial conflicts of interest [38] and market exclusivity and artificial inflation of drug prices [39] emerge, but these are not primary concerns.

Figure 3 compares the frequency with which articles from different disciplines address the social and ethical implications of AI-based facial technologies in medicine and healthcare. A broad and cross-disciplinary focus is evident on safety/technical robustness/clinical risk, privacy/confidentiality/surveillance, data governance and secondary use, and consent/autonomy. By contrast, dehumanisation and relational aspects of care receive relatively lower and more uneven attention across fields. The analysis further shows that each field accentuates distinct issues, with bioethics/philosophy/ethics and social sciences/STS/humanities offering the most cross-cutting coverage across the full range of concerns. In contrast, disciplines as computer science, engineering, AI and clinical fields appear to place less emphasis on issues of justice, stigma and accountability than other disciplinary domains. The data suggests the presence of a core set of topics that are shared by all fields but also indicates that the social and ethical concerns surrounding facial AI are multifaceted and influenced by the priorities and perspectives of each discipline.

## Discussion

Across the literature we reviewed, social and ethical debates about AI-based facial technologies in medicine and healthcare largely mirror the established agenda of AI-in-health ethics. The issues most consistently foregrounded – safety and clinical risk, privacy and data governance, and autonomy and consent – align closely with mainstream bioethical frameworks, including principlism [15, 25, 68, 69]. Safety and risk are typically framed through harm avoidance and acceptable risk–benefit trade-offs, invoking non-maleficence (and, in some accounts, beneficence). Similarly, sustained attention to privacy, confidentiality, secondary use, and stewardship reflects the ethical and regulatory significance of informational self-determination, reinforcing both autonomy and non-maleficence [70, 71]. Although justice is frequently cited, more developed engagements with social justice (distributive, epistemic, and structural) appear less prominent than the first three clusters of concerns [72–74]. Within the included literature, patient and public perspectives were also comparatively uncommon: trust and acceptability are often invoked, but less often grounded in systematic accounts of how patients, caregivers, or clinicians experience facial AI in clinical and quasi-clinical contexts [73, 75–77].

At the same time, our mapping suggests that treating facial AI as a generic instance of AI-in-health is ethically and socially incomplete. Facial technologies directly implicate personal identity and social recognition, appearance norms, stigma, and social sorting in ways that are especially salient when faces become health data. Across several application areas (rare-disease diagnosis via automated phenotyping, psychiatric and neurological assessment, aesthetic and reconstructive surgery, adherence monitoring, and public-health surveillance) facial AI operates in settings where people may already be vulnerable and visibly exposed, and where classificatory outputs can stabilise categories of “normal” and “pathological” faces or “appropriate” and “inappropriate” emotions and behaviours [10, 46, 57]. These dynamics may be read as extending medicalising logics into domains of identity and everyday presentation, albeit through new technical infrastructures [72]. While such concerns are not unique, since analogous issues have long been raised in bioethical debates on genetic information and labelling [77, 78] and AI-based physiognomic research [79], the face introduces a distinctive immediacy and visibility, with potential consequences for dignity, self-understanding, and social standing that are not always captured by generic AI-ethics framings.

A further contribution of this review lies in showing how ethical reasoning is patterned by disciplinary domain and study type. Different disciplines mobilise different ethical repertoires, and this pluralism is valuable; however, it also generates blind spots. Technical and clinical publications often approach ethics in instrumental terms (e.g. risk mitigation, workflow optimisation, professional standards, or compliance) while giving less sustained attention to power, inequality, stigma, and lived experience. Conversely, bioethics/philosophy/ethics, law/regulation, and social sciences/STS/humanities more often foregrounds social meaning and political stakes, but may be less specific about clinical constraints, implementation pathways, and design choices through which social and ethical harms become practically salient. These differences are also visible across study types: the philosophical articles in our review consistently offer in-depth analysis and are oriented toward facial recognition, whereas the empirical and implementation-oriented studies, most of which concern facial analysis, more often address social and ethical issues briefly or as adjuncts to a primary technical or clinical aim. Overall, ethical attention appears unevenly distributed across the two modalities: facial analysis, most closely tied to the interpretation of faces as sources of health information, tends to receive more limited ethical discussion, whereas more in-depth analysis is concentrated on the identification and surveillance concerns associated with facial recognition. In this context, it is plausible that ethical debate is partially shaped by what can be operationalised within data-driven research designs [80], potentially narrowing the space for anticipation, prudence, and reflexivity [81]. Yet, for AI-based facial technologies in medicine and healthcare, risks and responsibilities cut across these registers, be them algorithmic performance, legal rights, clinical relationships, lived identity, and social order. In our perspective this makes disciplinary compartmentalisation itself a social and ethical concern.

Finally, our findings suggest priorities for ethics scholarship and future research. One priority is to treat justice-related questions, particularly those linked to stigma, appearance norms, and socially consequential classification as central rather than residual. A second is to make explicit the political–economic infrastructures through which AI-based facial technologies are developed and deployed in medicine and healthcare, including commercial partnerships, incentives for data reuse, and cross-sectoral data flows. A third aspect relates to the fact that surveillance-linked applications, while not absent from the literature we reviewed, they are often framed instrumentally, leaving underexamined their implications for behavioural governance and interpretive authority when emotions or compliance are inferred from faces [82]. Addressing these gaps calls for interdisciplinary ethical approaches that move beyond checklist applications of generic principles toward context-sensitive analyses integrating justice broadly conceived [83]. This points to the value of empirical and participatory ethics capable of bringing patient and public perspectives into ethical evaluation and governance [73, 84, 85]. More generally, facial AI is a test case for richer models of “responsible AI” in medicine and healthcare, in which social meaning, institutional power, and lived experience are integral to ethical appraisal.

## Conclusion

This scoping review shows that social and ethical debates on AI-based facial technologies in medicine and healthcare are dominated by concerns that closely mirror the established agenda of AI-in-health ethics, particularly safety, privacy, data governance, and autonomy. While these issues are clearly important, their prominence risks obscuring other ethical and social dimensions that are especially salient when faces become health data.

Our findings also highlight how ethical analysis is shaped by disciplinary domain and study type. This disciplinary patterning contributes to blind spots, particularly regarding justice-related concerns and the lived experiences of patients and publics, which remain comparatively underexplored.

AI-based facial technologies provide a critical test case for more context-sensitive ethical frameworks that attend to social meaning, structural inequalities, and participatory forms of ethical evaluation and governance. Strengthening future research in this direction will be essential for ensuring that the development and deployment of facial AI in medicine and healthcare are ethically robust and socially responsive.

## Supplementary Information

Supplementary Material 1.

Supplementary Material 2.

Supplementary Material 3.

## Data Availability

The dataset supporting this scoping review is included as additional file. Full-text copies of the analysed publications are not provided as supplementary material, as some are subject to publisher copyright and licensing restrictions and should be accessed through the original publishers, journals, or databases. Additional information may be available from the corresponding author upon reasonable request.
