# Supplementary material for: Social and ethical implications of AI-based facial technologies in medicine and healthcare: a scoping review
Source: BMC Med Ethics. 2026 Jul 28;27:222. doi: 10.1186/s12910-026-01564-7 (PMC13693119; doi:10.1186/s12910-026-01564-7)
Supplement: Supplementary file 1 [file 12910_2026_1564_MOESM1_ESM.pdf]

Full data extraction tool

Variables	Brief description
Authors	Surname of first author, surname of second author, surname of third author, *et al.*
Year	Year of publication.
Type of paper	Whether the articles are philosophical papers or empirical studies. Additional 'other' category for articles which fall outside of these categories (specify).
Type of AI-based facial technology	Identification of the AI-based facial technology under analysis: facial recognition (identification/authentication); facial analysis (interpretation/inference); both facial recognition and facial analysis.
Functions in the healthcare context	Description of the main functions of facial AI analyzed in the healthcare context: risk prediction/prognosis; diagnosis/ screening; identify individuals; monitoring/telemedicine; triage/ workflow/administration; research-only tool (dataset/method development); behavioral intervention/therapy; other (specify).
Disciplinary domains	Identification of the two main disciplinary domains that support the argument in the article: clinical medicine & surgical specialties; psychiatry/psychology/neuroscience; public health/nursing/population health; computer science/engineering/AI; law/regulation/data protection; bioethics/philosophy/ethics; social sciences/STS/humanities.
Key ethical documents QUAL	Identification of the key ethical documents cited by the authors.
Key ethical documents QUANT	Record the presence (yes) or absence (no) of the following categories of documents: data protection and AI-governance frameworks; technical or clinical norms/guidelines; theoretical or philosophical approaches; reports, campaigns, media, documents from NGOs; institutional procedures and approvals.
Social and ethical implications QUAL	Notes on the authors' accounts of the social and ethical implications of AI-based facial technologies on medicine and healthcare.
Social and ethical implications QUANT	Record the presence (yes) or absence (no) of the following categories: bias / discrimination / inequity; privacy / confidentiality / surveillance; consent / autonomy; accountability / liability; stigma / labelling / stereotyping; dehumanisation / relational aspects of care; data governance & secondary use; safety / technical robustness / clinical risk / harm; trust / acceptability; justice (distributive, epistemic); human agency and oversight; transparency; other (specify).
Mode of presenting ethical and social issues	Whether the ethical and social issues are: identified in general terms, without detailed analysis; mentioned with some reflection; analyzed in-depth (conceptually, analytically or empirically).
